# Hexagonal and Square Patterned Silver Nanowires/PEDOT:PSS Composite Grids by Screen Printing for Uniformly Transparent Heaters

**DOI:** 10.3390/polym11030468

**Published:** 2019-03-12

**Authors:** Xin He, Gengzhe Shen, Ruibin Xu, Weijia Yang, Chi Zhang, Zhihao Liu, Bohua Chen, Junyan Liu, Mingxia Song

**Affiliations:** 1 School of Applied Physics and Materials, Wuyi University, Jiangmen 529020, China; shengzwyu@126.com (G.S.); xurbwyu@163.com (R.X.); yangweijia5377@126.com (W.Y.); ch.zhang@outlook.com (C.Z.); wlxylzh607@163.com (Z.L.); cbhmusuia@163.com (B.C.); liujunyanwyu@126.com (J.L.); 2 Collaborative Innovation Center of Atmospheric Environment and Equipment Technology, Jiangsu Key Laboratory of Atmospheric Environment Monitoring and Pollution Control, School of Environmental Science and Engineering, Nanjing University of Information Science & Technology, Nanjing 210044, China; smx839@163.com

**Keywords:** transparent conductive film, silver nanowires, PEDOT:PSS, regular grid, heater

## Abstract

Transparent conductive films with hexagonal and square patterns were fabricated on poly(ethylene terephthalate) (PET) substrates by screen printing technology utilizing a poly(3,4-ethylenedioxythiophene) poly(styrenesulfonate) (PEDOT:PSS) and silver nanowire (Ag NWs) composite ink. The printing parameters—mesh number, printing layer, mass ratio of PEDOT:PSS to Ag NWs and pattern shape—have a significant influence on the photoelectric properties of the composite films. The screen mesh with a mesh number of 200 possesses a suitable mesh size of 74 µm for printing clear and integrated grids with high transparency. With an increase in the printing layer and a decrease in the mass ratio of PEDOT:PSS to Ag NWs, the transmittance and resistance of the printed grids both decreased. When the printing layer is 1, the transmittance and resistance are 85.6% and 2.23 kΩ for the hexagonal grid and 77.3% and 8.78 kΩ for the square grid, indicating that the more compact arrangement of square grids reduces the transmittance, and the greater number of connections of the square grid increases the resistance. Therefore, it is believed that improved photoelectric properties of transparent electrodes could be obtained by designing a printing pattern with optimized printing parameters. Additionally, the Ag NWs/PEDOT:PSS composite films with hexagonal and square patterns exhibit high transparency and good uniformity, suggesting promising applications in large-area and uniform heaters.

## 1. Introduction

In recent years, transparent and conducting films have been widely used in emerging optoelectronic devices such as touch screens, lighting and display panels, solar cells, and wearable electronic devices [1,2,3,4,5]. At present, conventional transparent conductive materials include indium tin oxide (ITO) [6], aluminum-doped zinc oxide (AZO) [7] and fluoride-doped tin oxide (FTO) [8], which possess excellent photoelectric properties. However, there are several issues limiting their further application in next-generation optoelectronic devices. For example, in order to prepare the films on flexible substrates, low-temperature processes are required, which usually causes incomplete crystallization of nanoparticles, increased film defects and strong grain boundary scattering. This results in the decline of the photoelectric properties of these thin films. In addition, the brittleness and high cost also impede their application [9].

Recently, researchers have developed several flexible and transparent materials to replace oxide electrodes, including graphene [10], carbon nanotubes [11], conductive polymers [12], MXenes [13], metal nanowires [14] and metal meshes [15]. Among these materials, metal mesh displays excellent photoelectric properties and high uniformity due to the ordered electronic transport pathways. Therefore, metal mesh is regarded as a promising candidate for emerging transparent conductive electrodes (TCEs).

The fabrication methods of metal meshes on flexible substrates typically include laser sintering [16], ink filling [17], templating [18], inkjet printing [19,20,21,22], photolithography [23] and nano-imprint lithography [24]. Ko et al. prepared an orderly Ag grid with a line width of 10–15 µm, transmittance of 85%, and sheet resistance of 30 Ω/sq using a laser sintering method [16]. Chen et al. fabricated the embedded Ag grid and PEDOT:PSS composite TCE on PET substrate using an ink filling method [17]. The film exhibited a transmittance of 85% and a sheet resistance of 0.5 Ω/sq was successfully applied in solar cells. Gao et al. used the TiO_2_ gel cracks as the template to fabricate self-assembly Ag grids with a transmittance of 88% and a sheet resistance of 10 Ω/sq [18]. Zhang et al. combined the inkjet printing method and the coffee ring effect to prepare an Ag mesh with a line width of 5–10 µm, a transmittance of 93.6%, and a sheet resistance of 30 Ω/sq [20]. They further improved the robust adhesion of metal mesh to substrate by hydrophilic treatment without decreasing the photoelectric performance of the TCE film [22]. Huang et al. used a lithography method to fabricate TCE based on hierarchical metal mesh with a transmittance of 83.1% and a sheet resistance of 9.8 Ω/sq [23]. Yi et al. prepared an Ag grid with a moth-eye nanostructure utilizing nano-imprinting technology, presenting a transmittance of 85.9% and a sheet resistance of 22.8 Ω/sq [24].

TCEs based on metal meshes have excellent photoelectric properties, indicating great application prospects in flexible photoelectric devices. However, there are still several problems in the above fabrication techniques of metal meshes. For instance, the size of used the nanomaterials is restricted by the size of the nozzle in the inkjet printing method, and a further sintering process is required for connecting isolated nanoparticles. The photolithography method requires a complicated device with high-cost fabrication [25]. The nano-imprinting and ink-filling method requires precision templates and grooves, which are usually prepared by means of intricate processes.

Therefore, a rapid, environmentally friendly and cost-effective method is required to fabricate TCE with a designed pattern. In this work, uniform grids with hexagonal and square patterns are prepared by a facile screen-printing process, which is a technique possessing scalable production, low cost and high efficiency [2,26,27]. Additionally, its preparation process at room temperature is compatible with flexible organic substrates with low melting point. Ag NWs dispersed in alcohol could be utilized to print on the flexible substrate to fabricate TCE with regular grids. However, the as-obtained ink is very easy to diffuse, consequently destroying the designed pattern. Herein, we introduced the conductive polymer PEDOT:PSS into the Ag NW ink to produce the composite printing-ink [28,29]. The clear and regular grids could be fabricated using the ink, and the adhesion strength of the mesh to the substrate was also greatly improved. Furthermore, the effects of the printing mesh number, printing layer, mass ratio of PEDOT:PSS to Ag NWs and pattern shape on the photoelectric properties of the TCEs were carefully investigated. The films show promise for application in transparent, uniform, low-cost and large-area heaters. The heating performances of the meshes with hexagonal and square pattern were also discussed.

## 2. Experimental Sections

### 2.1. Synthesis of Ag NWs

Ag NWs were prepared by a hydrothermal method as described elsewhere [30]. Firstly, 0.7218 g glucose, 0.5414 g silver nitrate and 0.2404 g ferric sulfate were dissolved in 100 mL deionized water at room temperature. The reactants were adequately stirred until a light-yellow solution appeared, followed by the addition of 4.5 g poly(vinyl pyrrolidone) (PVP, K30). After the PVP powders were completely dissolved, 40 mL resultant solution transferred into an autoclave, which was then sealed and heated at 180 °C for 9 h. After reaction, a gray-green precipitate was washed by centrifugations with ethanol for several times, and then was filtered for 2–3 times to obtain the Ag NWs.

### 2.2. Preparation of Ag NWs/PEDOT:PSS Composite Conductive Ink

A 10 wt% dimethyl sulfoxide (DMSO) was introduced into 0.3 g PEDOT:PSS (Orgacon, EL-P3145, Agfa, Mortsel, Belgium) solution at room temperature to improve the ink conductivity. After ultrasonic oscillation for 60 min, the homogeneous PEDOT:PSS ink with good conductivity was obtained. Following this, the dried Ag NWs were added into the PEDOT:PSS ink to generate composite inks with the assistance of magnetic stirring at 50 °C. Ag NW concentrations were varied in the range of 4–20 mg/mL. The viscosity of the inks with the mass ratio of PEDOT:PSS to Ag NWs of 1:0.6, 1:0.8, 1:1 and 1:1.2 are 1270, 2260, 2476 and 3650 mpas, respectively.

### 2.3. Fabrication of Ag NWs/PEDOT:PSS Composite Grid

**Figure 1:**
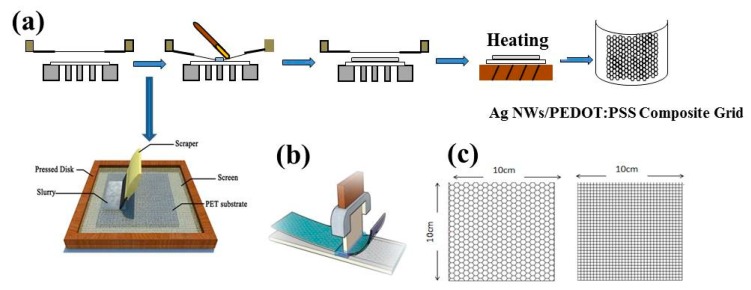
Schematic illustration of the screen-printed process of Ag NWs/PEDOT:PSS composite grids.

Ag NWs/PEDOT:PSS composite grids with hexagonal and square patterns were fabricated by a screen-printing method. Before printing, the PET substrate was washed with deionized water and ethanol with the assistance of ultrasonic treatment for 15 min to remove the dust, grease and other impurities on the substrate surface. Figure 1 shows the schematic illustration of the fabrication process of the composite grids. The detailed procedure is described as follows. Firstly, the substrate was adsorbed onto the surface of printing plate by a mechanical pump. The distance between the designed printing mesh and the substrate is maintained at 4 mm. Following this, the Ag NWs/PEDOT:PSS composite ink was dropped onto the mesh with an area of 10 cm × 10 cm, and the squeegee fleetly was pushed across the mesh with angle of approximate 45°. The printed films were finally dried at 70 °C for 20 min. After printing, the films were laminated using a mechanical pressure of 15 MPa for 3 min in order to enhance the contact of the Ag NWs and PEDOT:PSS. The resultant Ag NWs/PEDOT:PSS composite grids with certain patterns and good photoelectrical properties were finally obtained. The side length of each designed pattern is 3 mm, and the line width is 300 µm.

### 2.4. Characterization

The compositions of the composite grids were characterized using an X-ray diffractometer (XRD) (X’pert Pro MFD, Panalytical, the Netherlands) with a Cu Kα radiation (λ = 1.54178 Ǻ). The surface morphologies of transparent films were observed using a field emission scanning electron microscope (FE-SEM) (Zeiss Sigma 500, Germany) and optical microscope (DVM6A, Leica, Singapore). The transmittances were recorded using a UV-Vis spectrophotometer (UV2550, Shimadzu, Japan) and the reference spectrum was evaluated from air. The resistances of the films were evaluated using a digital multimeter (VC890C+, Victor, China). The temperature and heat distributions of the film were measured using an IR thermal imager (Ti32, Fluke, USA).

## 3. Results and Discussion

**Figure 2:**
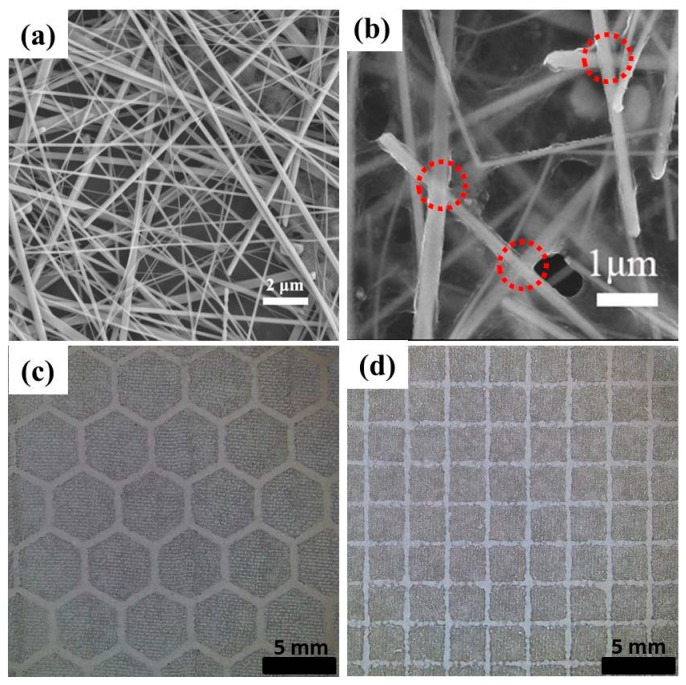
SEM image of the Ag NWs dispersed in ethanol (**a**) and Ag NWs/PEDOT:PSS composite film (**b**); Optical micrograph of the printed Ag NWs/PEDOT:PSS composite grids with hexagonal (**c**) and square patterns (**d**).

Figure 2a shows SEM image of the Ag NWs dispersed in ethanol, suggesting the generation of one-dimensional nanostructures. The average diameter of the Ag NWs is approximately 150 nm, and the lengths are up to dozens of micrometers. In a previous publication [30], we reported that the long Ag NWs of several hundred micrometers could form an effective electron percolation network on the substrate. However, very long Ag NWs are not required in this work, because they are hard to get through the mesh holes during the printing process. The size of the used screen mesh should match with the length of the prepared NWs. Moreover, the mesh number can also determine the pattern integrity. Thus, we used three screen meshes with mesh numbers of 100, 200 and 300 to print the composite grids for comparison.

When the mesh number is 100 with a large mesh size of 150 µm, although the Ag NWs are easy to get through the mesh holes, the composite conductive ink also rapidly diffuses on the substrate, leading to the generation of a deviant pattern. When using screen mesh with a mesh number of 300 (mesh size is 48 µm), the small mesh size limited the passage of conductive ink through the mesh holes. Thus, a discontinuous pattern was formed, which weakened the photoelectric properties of the composite grids. Consequently, the screen mesh with mesh number of 200 has a suitable mesh size of 74 µm, which favors the passage of conductive ink and the printing of an integrated pattern.

Figure 2b displays the SEM image of Ag NWs/PEDOT:PSS composite film, revealing the uniform distribution of NWs mixed with the polymer. The mechanical lamination on the film makes a tight contact between NWs and the conductive polymer. Meanwhile, the connections among the NWs (shown in the red circles in Figure 2b) also become tight under lamination, leading to a decrease in film resistance [30]. The close contact of the NWs can greatly enhance the conductivity of the network. Additionally, the full coverage of polymer on the NWs isolates them from the air and prevents the oxidation of the NWs. Simultaneously, the polymer surrounding the NWs can provide good adhesion to the PET substrate. The optical micrograph of the grids with hexagonal and square pattern printed with the mesh number of 200 are shown in Figure 2c,d, demonstrating that relatively clear and connected grids can be obtained using the conductive ink.

**Figure 3:**
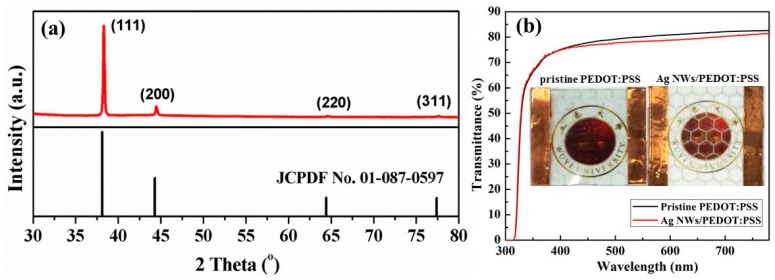
(**a**) XRD pattern of the composite grid with hexagonal pattern; (**b**) Optical transmittance spectra of the pristine PEDOT:PSS and Ag NWs/PEDOT:PSS hexagonal grid; the insets are the corresponding comparison photographs.

An XRD test was applied to confirm the phase composition of the as-obtained grids. Figure 3a presents the XRD pattern of the Ag NWs/PEDOT:PSS grid with a hexagonal pattern. Four diffraction peaks observed at 38.3°, 44.5°, 64.7° and 77.7° can be assigned to the (111), (200), (220) and (311) reflections of metallic silver (JCPDF No. 01-087-0597), respectively. No other peaks could be observed, indicating the absence of impurities in the composite grid. Figure 3b displays optical transmittance spectra of the pristine PEDOT:PSS and Ag NWs/PEDOT:PSS grid printed with the mesh number of 200. The mass ratio of PEDOT:PSS to Ag NWs is 1:1, and the four layers are printed for both samples. The average transmittance of the pristine PEDOT:PSS and composite film at 550 nm is 80.1% and 78.4%, respectively, indicating that the film transparency was maintained well after the addition of Ag NWs to the ink. This can also be confirmed by the comparison photography of two films, as seen in the inset of Figure 3b.

**Figure 4:**
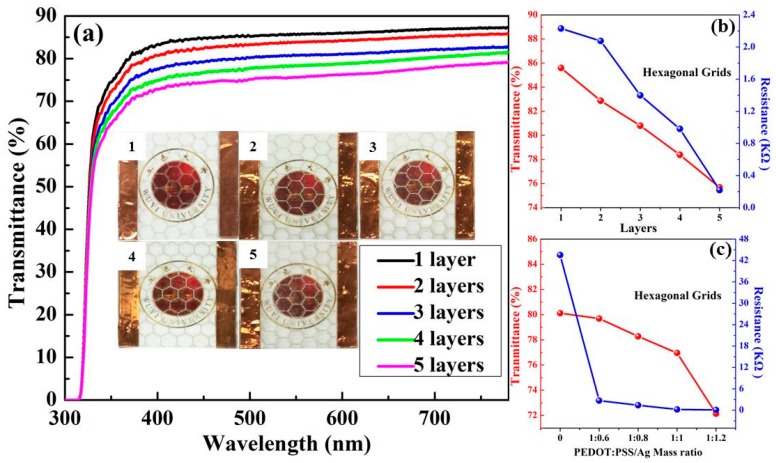
(**a**) Optical transmittance spectra of the Ag NWs/PEDOT:PSS hexagonal grids printed with 1 to 5 layers; the insets are photographs of corresponding grids. Variation of the transmittance at 550 nm and resistance of hexagonal grids with (**b**) the printed layer and (**c**) the mass ratio of PEDOT:PSS to Ag NWs.

The pattern shape is another factor determining the film performance. Therefore, the hexagon and square grids were printed for comparison. Figure 4a depicts optical transmittance spectra of the Ag NWs/PEDOT:PSS hexagonal grids printed with 1 to 5 layers. The film thickness can reach several micrometers. With the printing layer increased, the pattern of each layer would not be completely overlapped. Thus, the deviation among the printing patterns gradually increased, leading to the decrease of the transparency and conductivity uniformity of the grid. Resultantly, the printing layer of the grids is controlled to be 5 at most. The grid transmittances at 550 nm were 85.6%, 83.9%, 80.8%, 78.4% and 75.7% as the printing layer correspondingly increased from 1 to 5. The insets of Figure 4a are photographs of the grids printed with various layers, suggesting that the grid lines become easier to recognize, and all composite films are able to maintain high transparency. Figure 4b describes the transmittance and resistance of the composite films as a function of the printing layer. The transmittance and resistance both decreased with the increase of printing layer. The resistance of the composite film correspondingly is dropped from 2.23 to 0.22 kΩ, along with the printing layer increasing from 1 to 5. It should be clarified that the film conductivity is evaluated by the resistance between two sides of the film rather than sheet resistance in this work. This is because the line width of the grids using the screen-printing technology is relatively large, and the average conductivities of the grids are better characterized by the resistance than by sheet resistance.

Based on the above discussion, the film transmittance and resistance are dependent on the mesh number and printing layer. Additionally, the mass ratio of polymer to NWs is also considered. The changes in transmittance and resistance of the composite films with various mass ratios of PEDOT:PSS to Ag NWs are displayed in Figure 4c. All films are printed with the mesh number of 200 and layer of 5. The pristine PEDOT:PSS polymer film with the transmittance of 80.1% and resistance of 43.50 kΩ is provided for comparison. The mass ratio of PEDOT:PSS to Ag NWs is controlled at 1:0.6, 1:0.8, 1:1 and 1:1.2. With the contents of Ag NWs gradually increasing, the grid transmittance reduced from 79.7% to78.3%, 77.0% and 72.1%, respectively. Although the amount of Ag NWs has little effect on the film transparency, it greatly alters the grid conductivity. The film resistance decreased substantially from 43.50 to 2.68 kΩ once a small amount of Ag NWs was added due to the excellent conductivity of metal. Moreover, the resistance could decrease down to 78 Ω when the mass ratio is 1:1.2. It is worth noting that when the amount of Ag NWs was further elevated, the NWs would not homogeneously disperse in the ink, which is not conducive to printing an integrated grid.

**Figure 5:**
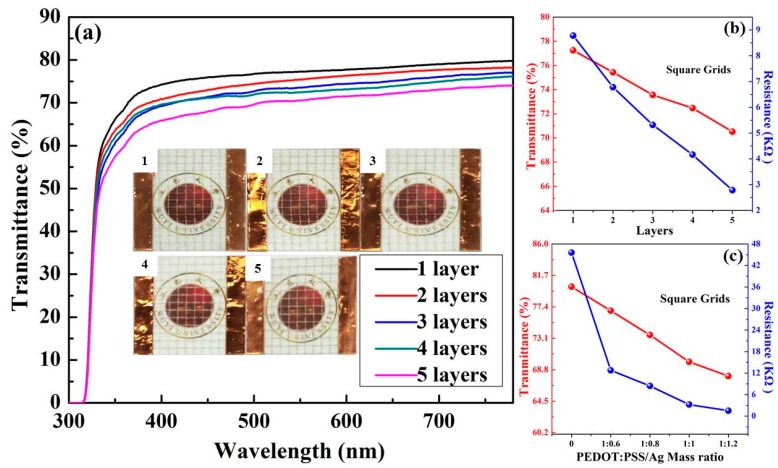
(**a**) Optical transmittance spectra of the Ag NWs/PEDOT:PSS square grids printed with 1 to 5 layers; the insets are photographs of corresponding grids. Variation of the transmittance at 550 nm and resistance of square grids with (**b**) the printed layer and (**c**) the mass ratio of PEDOT:PSS to Ag NWs.

Figure 5a presents the optical transmittance spectra of the Ag NWs/PEDOT:PSS composite grids with a square pattern. The insets of Figure 5a are photographs of corresponding grids, revealing high transparency of the films. With the printing layer increased from 1 to 5, the transmittance value at 550 nm is decreased from 77.3% to 70.5%, with the resistance of the composite films decreasing from 8.78 to 2.78 kΩ, as displayed in Figure 5b. In comparison with the hexagonal grids, the square shape exhibited a slight decrease in transmittance due to its more compact arrangement. Although the two patterns have the same side length, the printed grids display different blank areas, resulting in the transmittance difference between the two grids. In addition, the square grid has greater connection numbers of lines than the hexagonal grid. The connections can provide more barrier of electron transport. Thus, the square grids possess relatively higher resistances.

Figure 5c illustrates the relationship of the transmittance at 550 nm and resistance of square grids with the mass ratio of PEDOT:PSS to Ag NWs. When the mass ratio of PEDOT:PSS to Ag NWs is 1:0.6, 1:0.8, 1:1 and 1:1.2, the grid transmittance is 76.9%, 73.6%, 69.9% and 67.9%, with corresponding resistance of 12.78, 8.42, 3.24 and 1.52 kΩ. For comparison, pristine PEDOT:PSS film with square pattern exhibited a transmittance of 80.2% and resistance of 45.60 kΩ, suggesting similar photoelectric property with that of the hexagonal grid. Therefore, the conductivity of the Ag NWs/PEDOT:PSS composite film is mainly determined by the addition of Ag NWs and the connection number of the grid lines.

The change trend of transmittance and resistance with the printing layer and mass ratio of PEDOT:PSS to Ag NWs is the same when comparing the photoelectric properties for two grids. However, the grids with hexagonal pattern displayed a higher transparency and conductivity than the grids with square pattern using the same printing layer and concentrated ink, revealing that effective electronic conducting pathway in the grid could be obtained by designing the pattern shape and optimizing the printing parameters. Furthermore, we compared the photoelectric properties of the Ag NWs/PEDOT:PSS composite films with and without using the patterned meshes, as shown in Figure S1 and Table S1. For a given printing layer or resistance, a higher transmittance of the composite films using the patterns can be achieved.

Furthermore, we applied the composite grids in a transparent heater in order to better understand their application performance. The heating performances of the grids were measured using a two-terminal side contact configuration. The applied DC voltage was supplied by a power supply to the heater through two copper conductive tapes pasted at the film edges. The hexagonal and square grids with the transmittance of 77.0% and 69.9%, and the resistance of 0.22 and 3.24 kΩ were chosen for the application in transparent heaters. In the case of two samples, the printing layer was 5, and the mass ratio of PEDOT:PSS to Ag NWs was 1:1.

**Figure 6:**
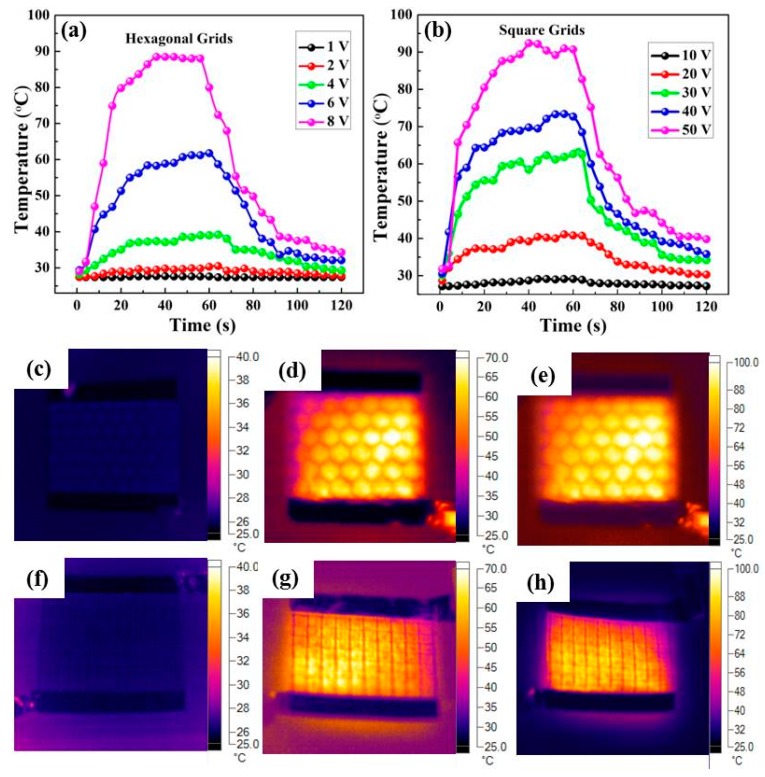
Evolution of generated temperature of the Ag NWs/PEDOT:PSS composite film with (**a**) hexagonal pattern at varied voltage from 1 to 8 V, and (**b**) with square pattern at varied voltage from 10 to 50 V; Infrared thermal images of the hexagonal grid using applied voltage of (**c**) 1 V, (**d**) 6 V and (**e**) 8 V; Infrared thermal images of the square grid using applied voltage of (**f**) 10 V, (**g**) 40 V and (**h**) 50 V.

Figure 6a,b depict the temperature profiles of the hexagonal and square grids, which are plotted with respect to various voltages. The generated temperature of the film heater increased as the voltage increased. When the applied potential on the hexagonal grid increased from 1 to 8 V, the film heater rose in temperature from 27.2 to 88.5 °C. However, the square grid needs a higher voltage to heat to a comparable temperature. When the potential was set at 10, 20, 30, 40 and 50 V, the saturated temperature was 29.1, 40.1, 62.3, 73.3 and 92.4 °C, respectively. Figure 6c–e presents the typical infrared thermal images of the hexagonal grid at the voltage of 1, 6 and 8 V, while Figure 6f–h shows the infrared images of the square grid at the driving voltage of 10, 40 and 50 V. The heat distributions of the grids are uniform across the whole substrate, revealing excellent application prospects in large-scale and uniform heating. The hexagonal grid exhibited a lower driving potential than the square grid, which could be ascribed to its better conductivity.

**Figure 7:**
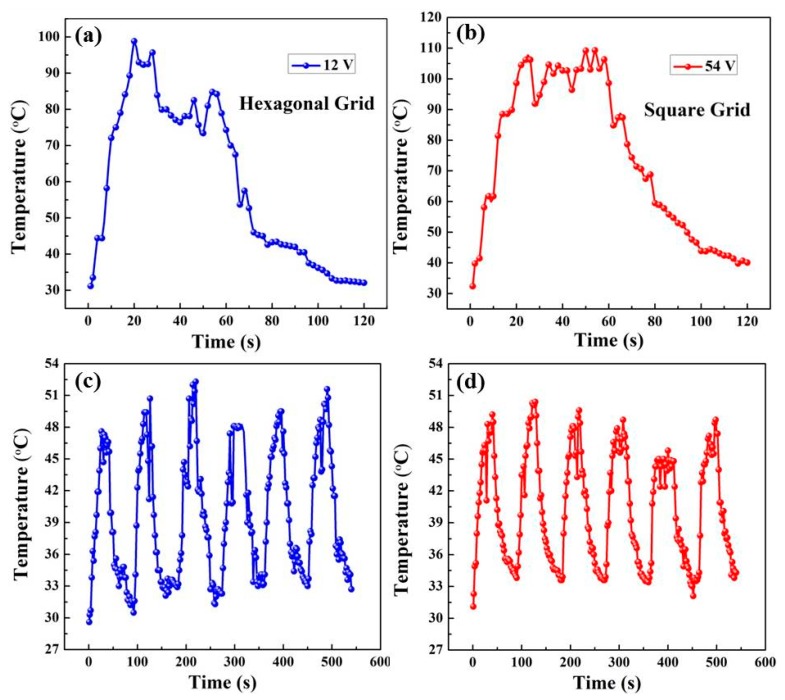
Evolution of generated temperature of the Ag NWs/PEDOT:PSS composite film with hexagonal and square pattern at (**a**) 12 V and (**b**) 54 V, respectively; On/off responses of the film heaters with hexagonal and square pattern at (**c**) 5 V and (**d**) 25 V, respectively.

The maximum heating temperature is determined by the driving voltage and melting point of the PET substrate, together. If the potential was further increased to 12 V on the hexagonal grid, the generated temperature rose to approximately 99 °C within 20 s. However, the conductive grid would be partially destroyed, and exhibited an unstable heating profile (Figure 7a), which was due to the generation of high heat at the NW junctions with high resistance under a large input voltage within a short period. Furthermore, when a potential of 54 V was applied on the square grid, it could be heated to 107 °C within 20 s, as displayed in Figure 7b. However, the high temperature exceeds the melting point of PET, resulting in the deformation of the substrate.

Figure 7c,d presents the on/off response of the film heaters with hexagonal and square grid, which was respectively characterized under driving voltage of 5 and 25 V. The heating and cooling time in each cycle was 40 and 50 s. The cycling curves of two grids indicated relatively stable temperature recoverability and saturation temperature of 50 °C. A long on/off response for the film heaters with hexagonal and square grid for 8 h was also conducted, with the stable recoverability indicating that the flexible heaters based on the Ag NWs/PEDOT:PSS composite grids can constantly rerun at a given potential for a long time.

Therefore, the Ag NWs/PEDOT:PSS composite films with hexagonal and square patterns could be fabricated by a scalable screen-printing technology and exhibited high transparency and good uniformity, suggesting great application prospects in large-area and uniform heaters. The photoelectric and heating performance of the films could be controlled by designing the film pattern and optimizing printing parameters.

## 4. Conclusions

In this work, we utilized the advantages of PEDOT:PSS and Ag NWs to screen-print transparent and conducting film with hexagonal and square patterns. The regular grids not only exhibit good adhesion to substrate, but also high transparency, uniformity and good conductivity. A clear and integrated grid can be printed with a mesh number of 200. The increase in the amount of Ag NWs in the ink and printing layer caused a slight decrease in the transmittance and a significant increase in the conductivity of the composite films. The heating performances of the grids with hexagonal and square patterns were compared, presenting uniform and fast response of heating. This investigation is expected to provide a guide for fabricating low-cost, large-scale and uniform TCE with a designed pattern.

## Supplementary Materials

The supplementary materials are available online at https://www.mdpi.com/2073-4360/11/3/468/s1.
