# Supplementary material for: Hexagonal and Square Patterned Silver Nanowires/PEDOT:PSS Composite Grids by Screen Printing for Uniformly Transparent Heaters
Source: Polymers (Basel). 2019 Mar 12;11(3):468. doi: 10.3390/polym11030468 (PMC6473526; doi:10.3390/polym11030468)
Supplement: Supplementary file 1 [file polymers-11-00468-s001.pdf]

Hexagonal and Square Patterned Silver Nanowires/PEDOT:PSS Composite Grids by Screen Printing for Uniformly Transparent Heaters

Xin He [1]*, Gengzhe Shen [1], Ruibin Xu[1], Weijia Yang[1], Chi Zhang[1], Zhihao Liu[1], Bohua Chen[1], Junyan Liu[1] and Mingxia Song[2]

[1] School of Applied Physics and Materials, Wuyi University, Jiangmen 529020, Guangdong Province, P.R. China;

[2] Collaborative Innovation Center of Atmospheric Environment and Equipment Technology, Jiangsu Key Laboratory of Atmospheric Environment Monitoring and Pollution Control, School of Environmental Science and Engineering, Nanjing University of Information Science & Technology, Nanjing, 210044, Jiangsu Province, P.R. China.

* Correspondence: hexinwyu@126.com; Tel.: +86-750-3296095

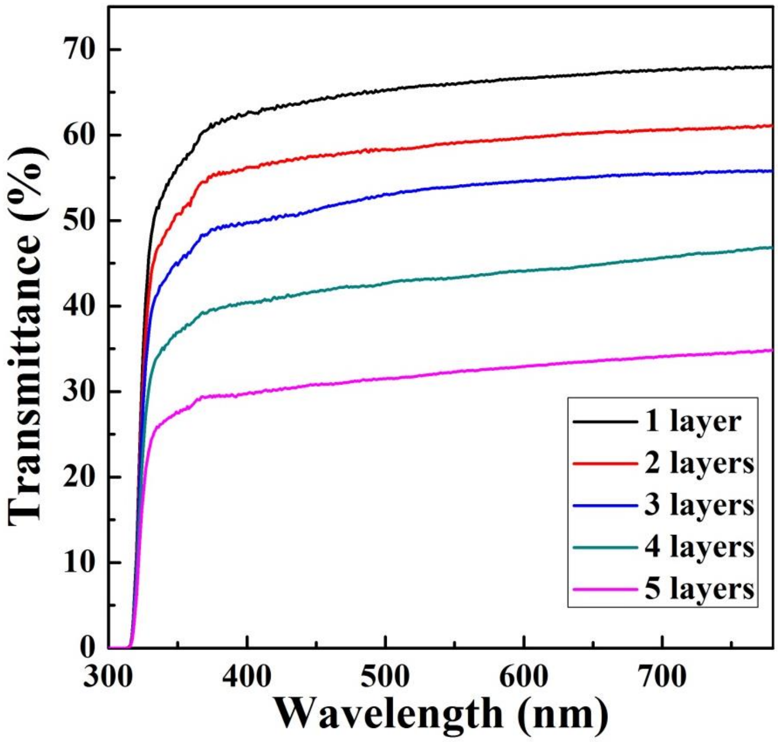

Figure S1 Optical transmittance spectra of the Ag NWs/PEDOT:PSS composite films without using patterned mesh, which were printed for 1 to 5 layers with the mass ratio of PEDOT:PSS to Ag NWs of 1:1.

Table S1 Comparison of the photoelectric properties of the Ag NWs/PEDOT:PSS composite films with and without using the patterned meshes.

Sample Name	T and R for 1 layer	T and R for 2 layers	T and R for 3 layers	T and R for 4 layers	T and R for 5 layers
**without pattern**	65.9%, 2.05 Ω	59.0%, 1.51 Ω	53.9%, 1.01 Ω	43.3%, 0.89 Ω	32.3%, 0.61 Ω
**hexagonal pattern**	85.6%, 2.23 Ω	83.9%, 2.08 Ω	80.8%, 1.40 Ω	78.4%, 0.98 Ω	75.7%, 0.22 Ω
**square pattern**	77.3%, 8.78 Ω	75.4%, 6.78 Ω	73.6%, 5.32 Ω	72.5%, 4.16 Ω	70.5%, 2.78 Ω
